# Supplementary material for: Model-based analysis uncovers mutations altering autophagy selectivity in human cancer
Source: Nat Commun. 2021 May 31;12:3258. doi: 10.1038/s41467-021-23539-5 (PMC8166871; doi:10.1038/s41467-021-23539-5)
Supplement: Supplementary file 1 — Supplementary Information [file 41467_2021_23539_MOESM1_ESM.pdf]

Supplementary Information

Model-based analysis uncovers mutations altering autophagy selectivity in human cancer

Zhu Han[1,†], Weizhi Zhang[2,†], Wanshan Ning[2], Chenwei Wang[2], Wankun Deng[2], Zhidan Li[1], Zehua Shang[1], Xiaofei Shen[3], Xiaohui Liu[4], Otto Baba[5], Tsuyoshi Morita[5], Lu Chen[1], Yu Xue[2,6,*], Da Jia[1,*]

[1]Key Laboratory of Birth Defects and Related Diseases of Women and Children, Department of Paediatrics, West China Second University Hospital, State Key Laboratory of Biotherapy and Collaborative Innovation Center of Biotherapy, Sichuan University, Chengdu 610041, China

[2]Key Laboratory of Molecular Biophysics of Ministry of Education, Hubei Bioinformatics and Molecular Imaging Key Laboratory, Center for Artificial Intelligence Biology, College of Life Science and Technology, Huazhong University of Science and Technology, Wuhan, Hubei 430074, China

[3]Hospital of Chengdu University of Traditional Chinese Medicine, Chengdu 610075, China

[4]School of Life Sciences, Tsinghua University, Beijing 100084, China

[5]Oral and Maxillofacial Anatomy, Tokushima University Graduate School, 3-18-15, Kuramoto-cho, Tokushima 770-8504, JAPAN

[6]Nanjing University Institute of Artificial Intelligence Biomedicine, Nanjing, Jiangsu 210031, China

[†]The first two authors contributed equally to this work.

*To whom correspondence should be addressed.

Yu Xue, E-mail: xueyu@hust.edu.cn; Da Jia, E-mail: Jiada@scu.edu.cn.

Contents

Supplementary Figures 4

Supplementary Note 1. Additional comparisons of pLIRm and other existing methods 20

Supplementary References..22

Supplementary Figures

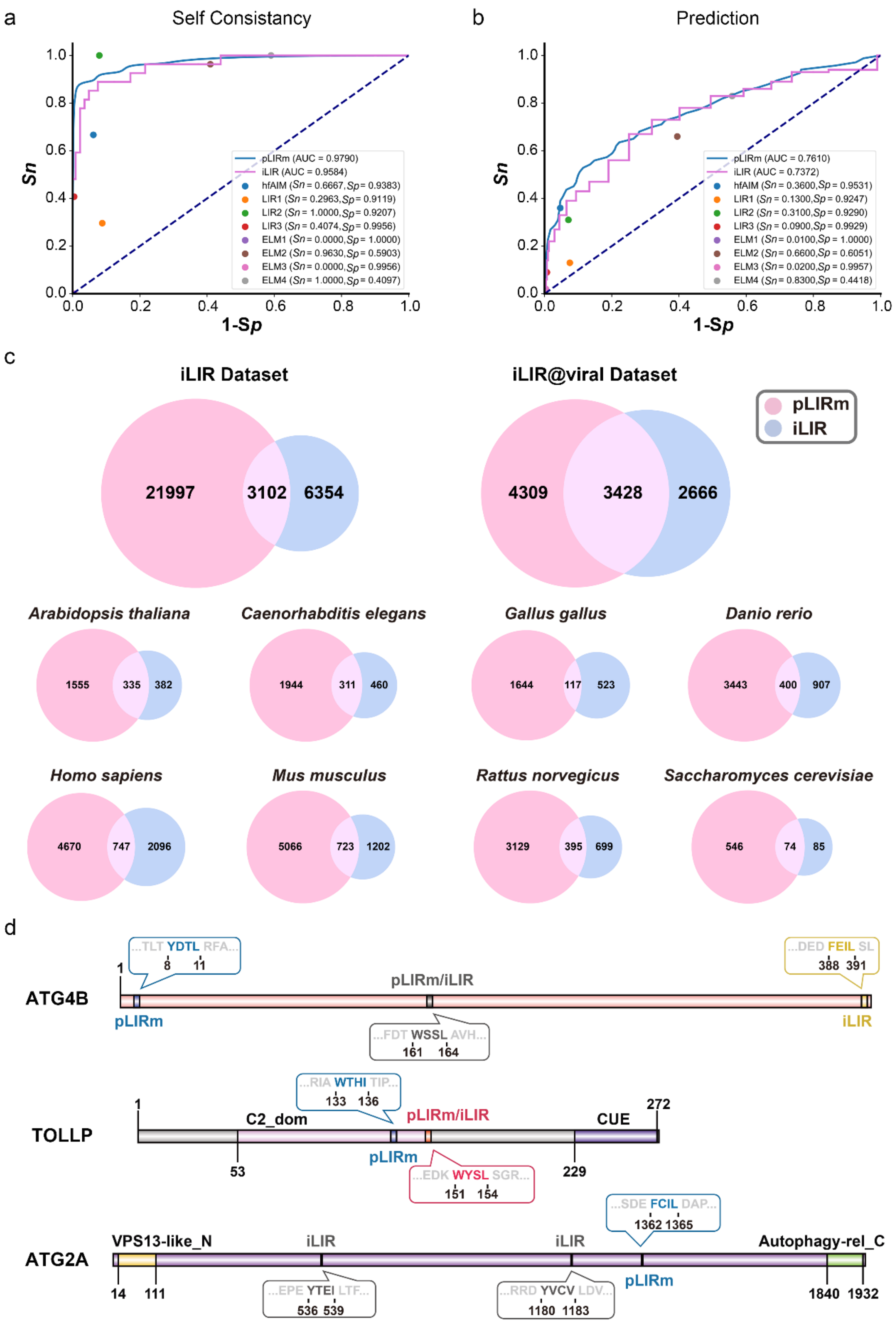

Supplementary Figure 1 | Comparison of pLIRm and other existing methods. **a**) The self-consistency validation is performed for pLIRm, iLIR, hfAIM, 3 reported LIR motifs and 4 sequence patterns in ELM using the same training dataset of iLIR [1]. **b**) An additional prediction is performed for pLIRm, iLIR, hfAIM, 3 reported LIR motifs and 4 sequence patterns in ELM on testing dataset not used for training iLIR. **c**) A moderate coverage between pLIRm and iLIR predictions on iLIR database and iLIR@viral database. **d**) Experimentally identified and potential LIRs predicted by pLIRm and iLIR on ATG4B, TOLLP and ATG2A.

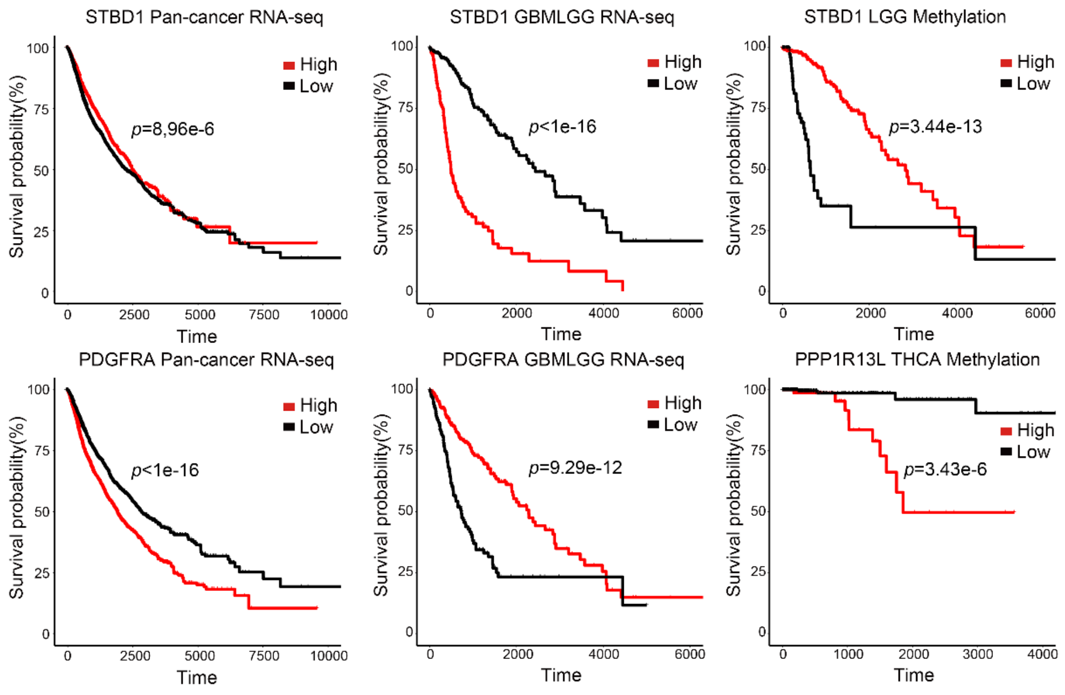

Supplementary Figure 2 | Controversial survival results of STBD1 and other oncogenes between pan-cancer and individual cancers. Higher mRNA expression level of STBD1 is associated with a longer survival rate in pan-cancer, but associated with a shorter survival rate in GBMLGG. Meanwhile, higher DNA methylation level of STBD1 is associated with a longer survival rate in LGG. For oncogenes, the higher mRNA expression level of PDGFRA in GBMLGG and lower DNA methylation level in THCA of PPP1R13L are significantly associated with a higher survival probability, exhibiting an opposite result against that in the pan-cancer level. The two-sided log-rank test is performed ($p < 0.05$), and the Kaplan-Meier survival curves are plotted by ggsurvplot.

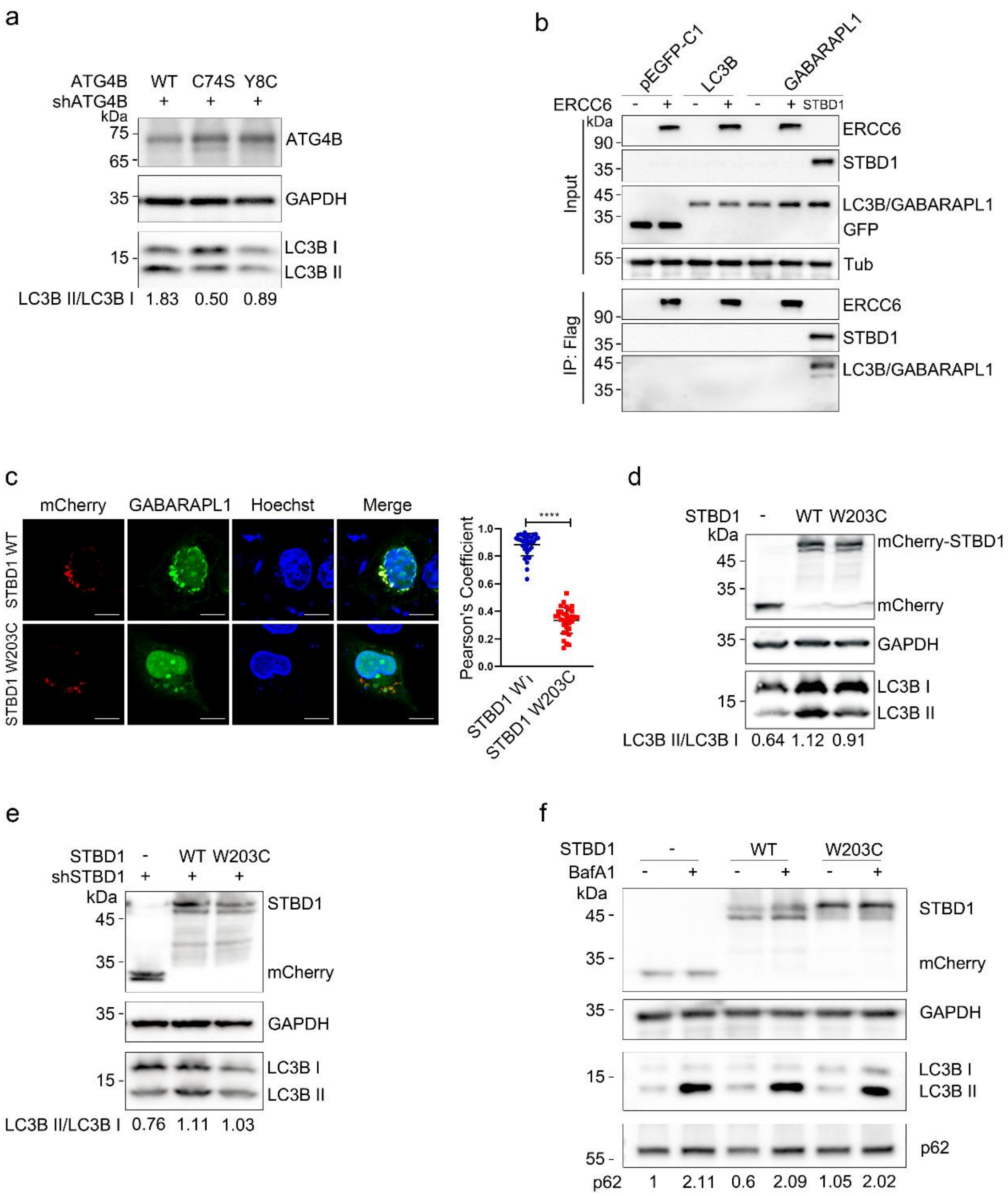

Supplementary Figure 3 | Mutations of ATG4B and STBD1 found in cancer patients impair their autophagic functions. a) HEK293T cells were transfected with shATG4B for 24 h, and then with shRNA-resistant constructs (ATG4B WT, C74S, Y8C) 48 h after the second transfection, cells were harvested and lysed for immunoblotting to determine the protein levels of ATG4B, LC3B I, LC3B II. The band of LC3B II was quantified by Image J and normalized to the level of LC3B I, and labeled below the blots. **b**) HEK293T cells were co-transfected with Flag-tagged

ERCC6 and GFP-tagged LC3B or GABARAPL1 for 24 h. Control cells (vector) were transfected with empty vector. One tenth of the cell lysate was prepared as input, and the rest was used for immunoprecipitation (IP) with anti-Flag Sepharose 4B gel followed by immunoblotting with indicated antibodies. **c**) Confocal immunofluorescence of HeLa cells co-transfected with mCherry-STBD1 WT or W203C, and GFP-tagged GABARAPL1 for 24 h. Nuclei were stained with Hoechst (blue). Images were captured using the Olympus FV-1000. Pearson's coefficients of STBD1 and GABARAPL1 was calculated using image J. Each dot represents the value of one cell. Statistical data were presented as mean ± SD. Statistical comparisons were performed using unpaired t-test. **** p <0.0001. Scale bar, 20 μm. **d**) HEK293T cells were transfected with mCherry N1, STBD1 WT or STBD1 W203C respectively. Forty-eight hours after transfection, cells were harvested and lysed for immunoblotting to determine the protein levels of STBD1, LC3B I, LC3B II. The band of LC3B II was quantified by Image J and normalized to the level of LC3B I, and labeled below the blots. **e**) HEK293T cells were transfected with shSTBD1 for 24 h, and then with shRNA-resistant constructs (STBD1 WT, W203C). Control cells were transfected with empty vector. Cells were harvested 48 h after the second transfection, and then lysed for immunoblotting to determine the protein levels of STBD1, LC3B I, LC3B II. The band of LC3B II was quantified by Image J and normalized to the level of LC3B I, and labeled below the blots. **f**) HEK293T cells were transfected with mCherry N1, STBD1 WT or STBD1 W203C respectively. Forty-eight hours after transfection, cells were treated with Bafilomycin A1 (BafA1, 200 nM) for 3 h, and then harvested and lysed for immunoblotting to determine the protein levels of STBD1, LC3B I, LC3B II and p62. The band of p62 was quantified by Image J and normalized to the level in control sample, and labeled below the blots. Experiments (a-f) were performed in triplicate.

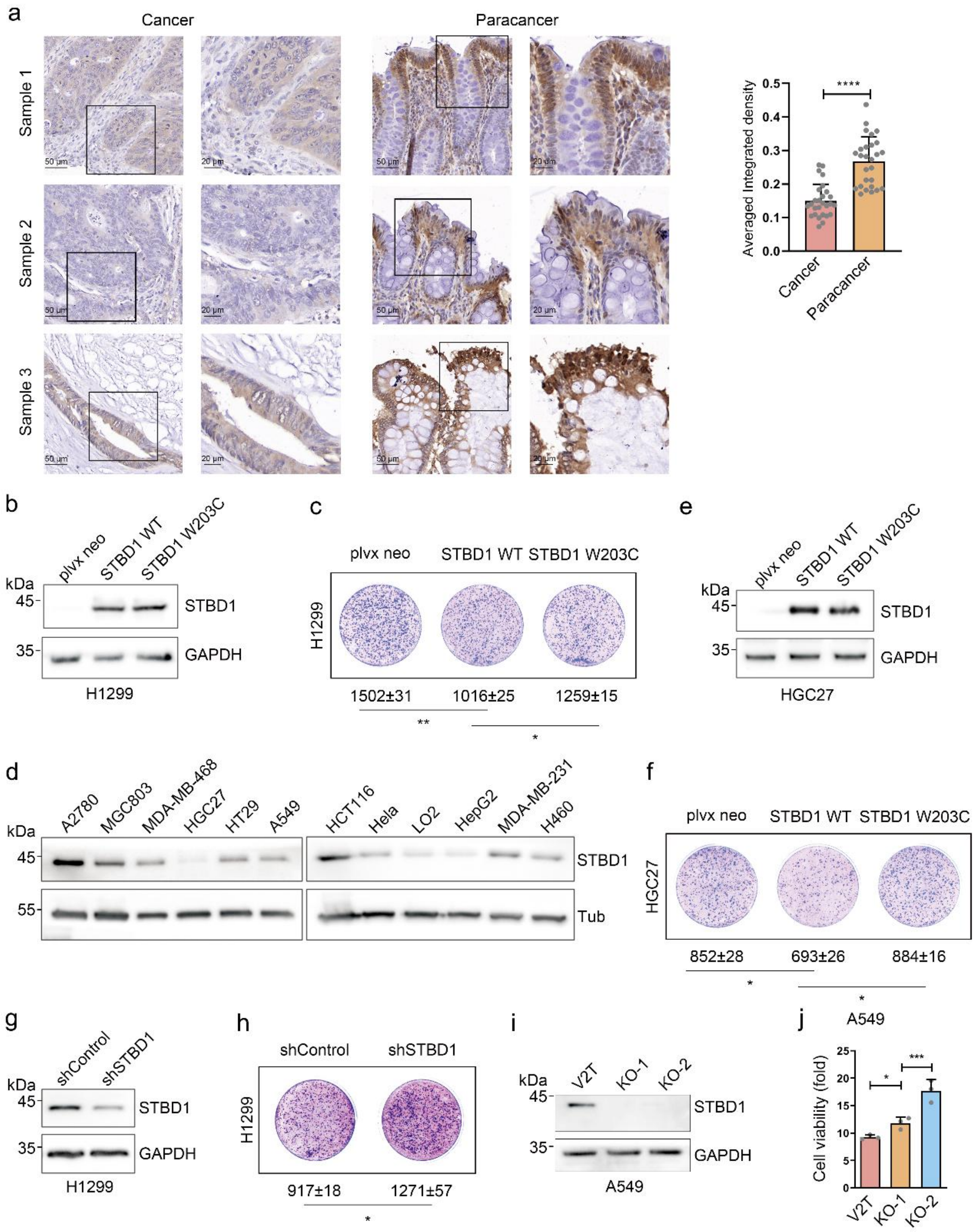

Supplementary Figure 4 | STBD1 WT, but not W203C, inhibits multiple cancer cell growth.

a) Representative images of IHC staining for STBD1 expression in the cancer and paracancer tissues of colon cancer patients (n=27). The expression of STBD1 in the cancer and paracancer tissues was determined by analyzing the optical density using Image J. **b**) Control H1299 cells (plvx neo) and H1299 cells stably-expressing STBD1 WT or W203C were lysed for

immunoblotting to determine the protein levels of STBD1 and GAPDH. **c**) Control H1299 cells (plvx neo) and H1299 cells stably-expressing STBD1 WT or W203C were cultured for 20 days, then stained by crystal violet. The number of colonies was analyzed using Image J. **d**) Protein expression of STBD1 in multiple cancer cell lines was determined by immunoblotting. **e**) Control HGC27 cells (plvx neo) and HGC27 cells stably-expressing STBD1 WT or W203C were lysed for immunoblotting to determine the protein levels of STBD1 and GAPDH. **f**) Control HGC27 cells (plvx neo) and HGC27 cells stably-expressing STBD1 WT or W203C were cultured for 20 days, then stained by crystal violet. The number of colonies was analyzed using Image J. **g**) Protein levels of STBD1, GAPDH in shControl and shSTBD1 H1299 cells were determined by immunoblotting. **h**) shControl and shSTBD1 H1299 cells were cultured for 20 days, then stained by crystal violet. The number of colonies was analyzed using Image J. **i**) Control A549 cells (empty vector) and two STBD1-knockout cell lines were lysed for immunoblotting to determine the protein levels of STBD1 and GAPDH. **j**) Control A549 cells (empty vector) and two *STBD1*-knockout cell lines were cultured for 84 h. The cell viability was then assessed using the MTT assay, and normalized to that of 0 h. Experiments (b-j) were performed in triplicate. (a-j) Statistical data are presented as mean ± SD. Statistical comparisons were performed using unpaired t-test. **** p <0.0001, *** p <0.001, ** p <0.01, * p <0.05.

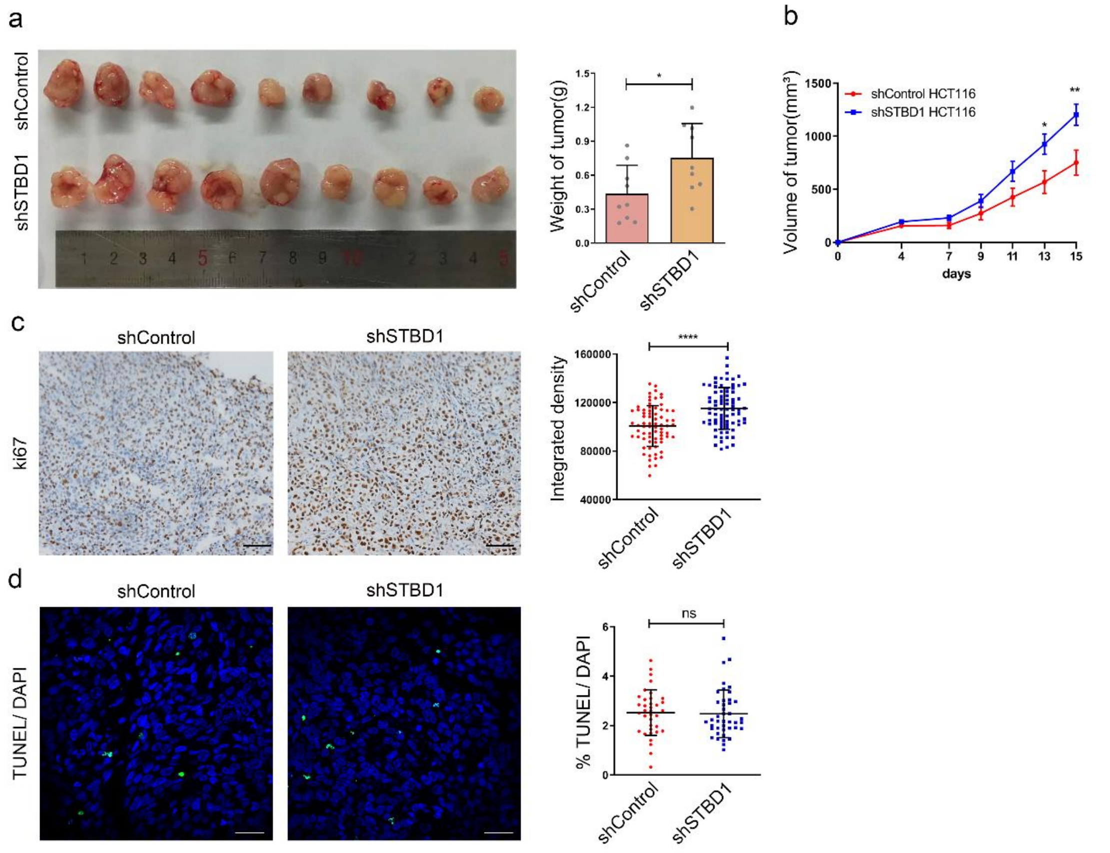

Supplementary Figure 5 | Depletion of STBD1 promotes tumor growth *in vivo.* **a**) Nude mice (n=9) were injected subcutaneously on both flanks with shControl or shSTBD1 HCT116 respectively. Images show the dissected tumors and tumor weights after 15 days. **b**) Tumor volume was measured over time after injection in mice as in (a). **c**) Examination of the proliferation marker Ki67 in tumor tissues by IHC. Images were captured using an Olympus microscope, and integrated density was determined using Image J. Scale bars, 100 μm. **d**) Detection of the apoptosis marker TUNEL in tumor tissues. The nuclei were labeled using DAPI. The images were captured using an Olympus microscope and the percentage of cells staining positively for TUNEL was determined using Image J. Scale bars, 50 μm. Statistical data are presented as mean ± SD. Statistical comparisons were performed using unpaired t-test. **** p <0.001, ** p <0.01, * p <0.05, ns (not significant) p >0.05.

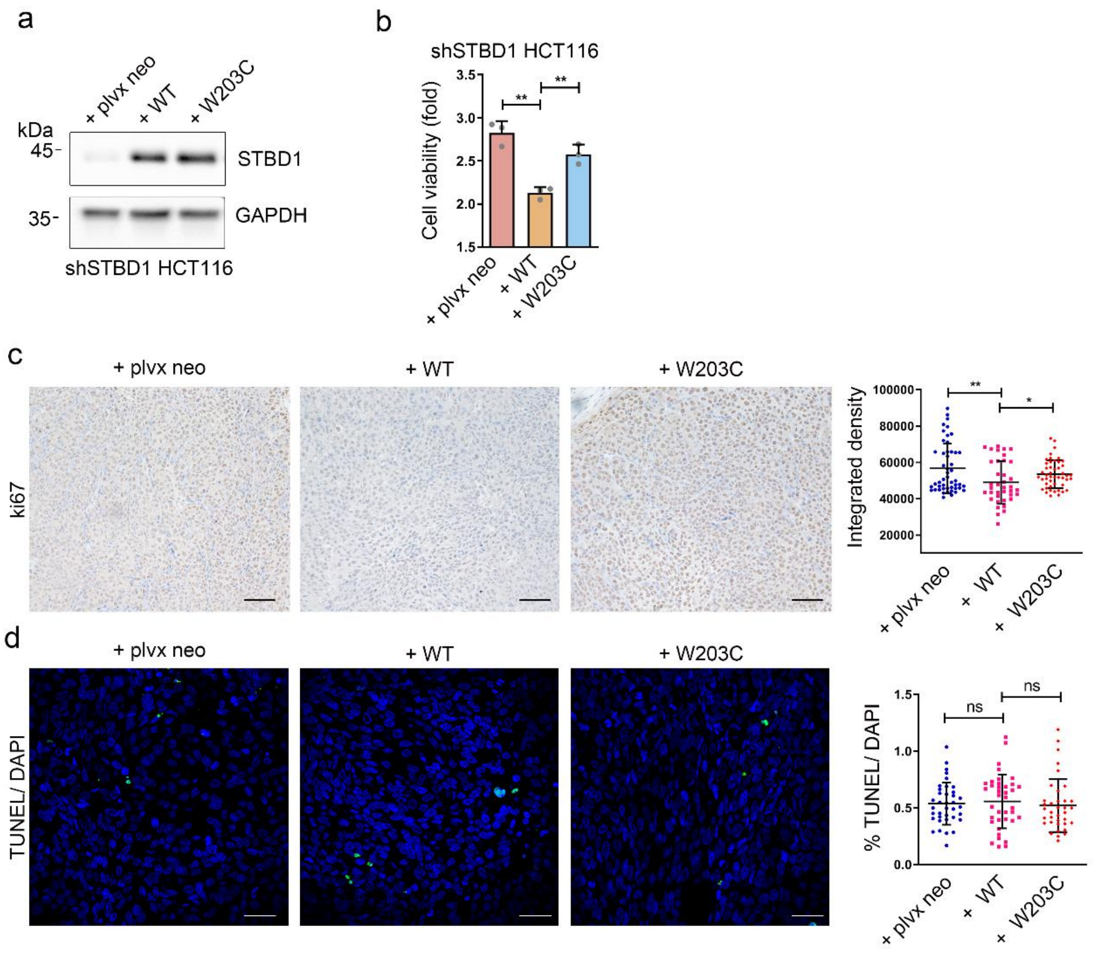

Supplementary Figure 6 | STBD1 WT inhibits tumor growth *in vivo* in comparison with control and STBD1 W203C. **a**) Protein expression of STBD1 and GAPDH in shSTBD1/plvx neo, shSTBD1/WT, and shSTBD1/W203C HCT116 cells. **b**) shSTBD1/plvx neo, shSTBD1/WT, and shSTBD1/W203C HCT116 cells were cultured for 48 h. The cell viability was assessed using the MTT assay and normalized to that of 0 h. **c**) Detection of the proliferation marker Ki67 in tumor tissues by IHC. Images were captured using an Olympus microscope, and integrated density was determined using Image J. Scale bars, 100 μm. **d**) Detection of the apoptosis marker TUNEL in tumor tissues. The nuclei were labeled using DAPI. Images were captured using an Olympus microscope, and the percentage of cells staining positively for TUNEL was determined using Image J. Scale bars, 50 μm. Statistical data are presented as mean ± SD. Statistical comparisons

were performed using unpaired t-test. ** $p < 0.01$, * $p < 0.05$, ns (not significant) $p > 0.05$.

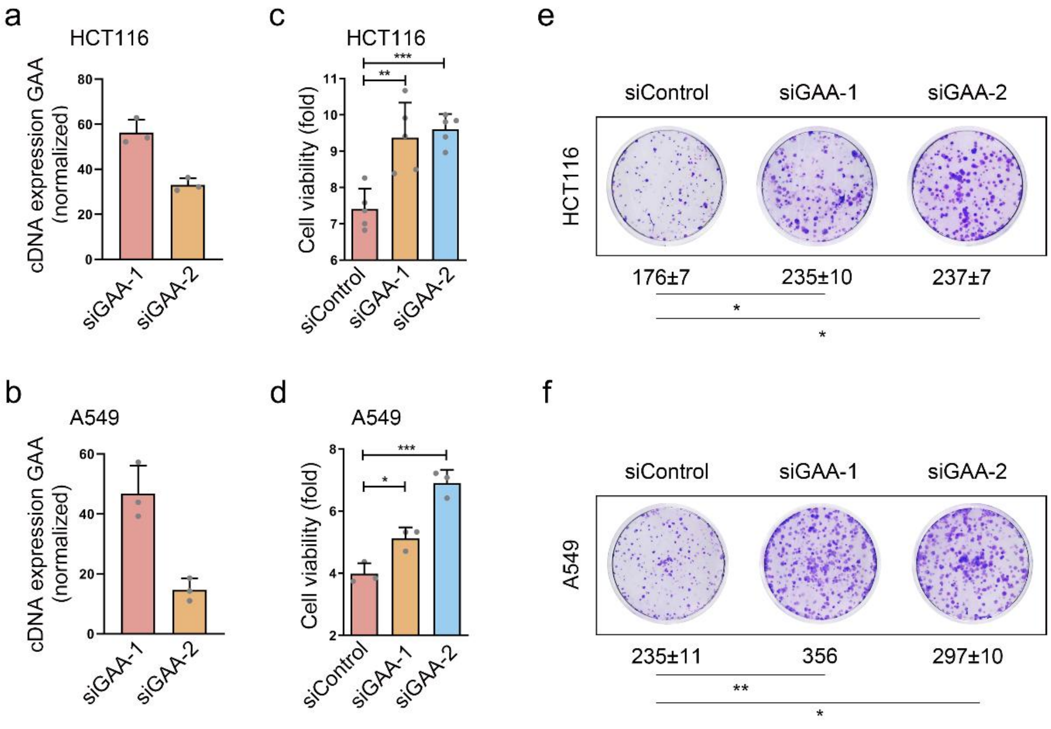

Supplementary Figure 7 | Depletion of lysosomal acid α-glucosidase (GAA) promotes multiple cancer cell growth. a/b) qRT-PCR analysis of lysosomal acid α-glucosidase (GAA) mRNA level after the transfection of HCT116/A549 cells with 2 different siRNA molecules (siGAA-1, siGAA-2). Knockdown efficiency was normalized using GAA mRNA level of HCT116 cells transfected with siControl. siControl = non-targeting control siRNA. **c/d**) HCT116/A549 cells were transfected with 3 different siRNA molecules (siControl, siGAA-1, siGAA-2) respectively and cultured for 72h. The cell viability was then assessed using the MTT assay and normalized to that of 0 h. **e/f**) HCT116/A549 cells were transfected with 3 different siRNA molecules (siControl, siGAA-1, siGAA-2) respectively every 3 days and cultured for 12 days, then stained by crystal violet. The number of colonies was analyzed using Image J. Experiments were performed in triplicate. Statistical data are presented as mean ± SD. Statistical comparisons were performed using unpaired t-test. *** $p < 0.001$, ** $p < 0.01$, * $p < 0.05$.

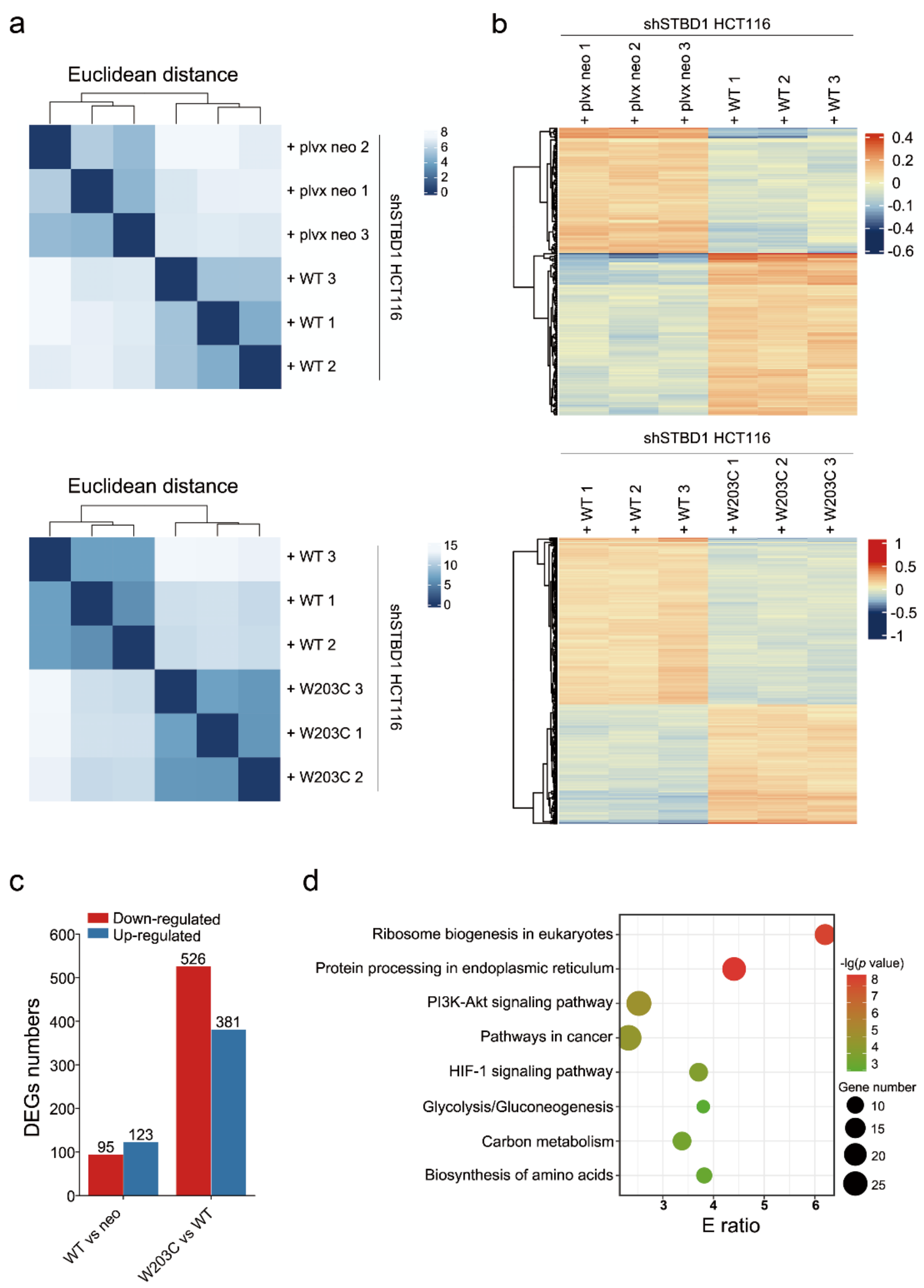

Supplementary Figure 8 | STBD1 W203C enhances expression of multiple genes in glycolysis.

a) Three biological replicates of shSTBD1/plvx neo, shSTBD1/WT, and shSTBD1/W203C HCT116 cells were clustered in RNA-seq. **b**) Heatmap of differentially expressed genes in shSTBD1/WT *vs.* shSTBD1/plvx neo cells (upper panel), and heatmap of differentially expressed genes in shSTBD1/W203C *vs.* shSTBD1/WT cells (lower panel). **c**) Numbers of down-regulated or up-regulated genes in differentially expressed genes. **d**) KEGG-based enrichment analysis of

biological pathway of differentially expressed genes in shSTBD1/W203C *vs.* shSTBD1/WT cells.

Shown are the pathways that are also enriched in the shSTBD1 cell.

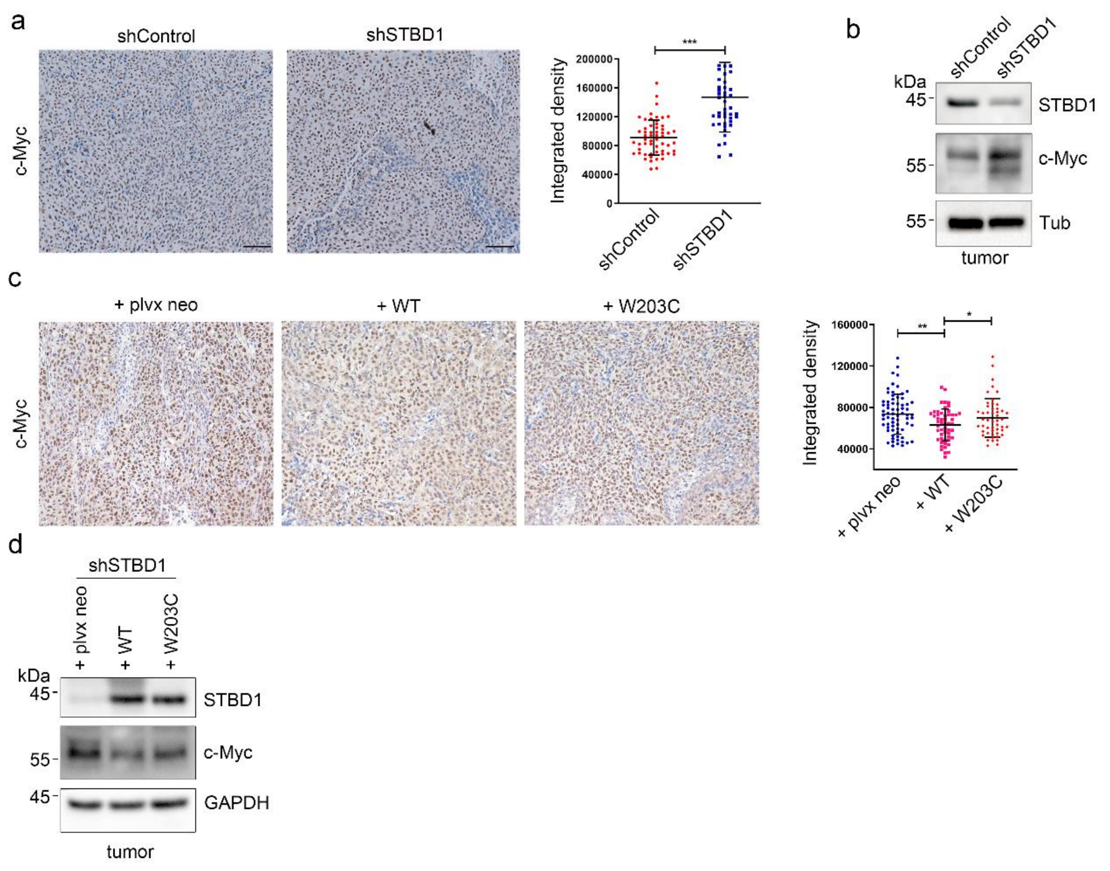

Supplementary Figure 9 | STBD1 WT, but not W203C, inhibits the expression of cancer hallmark gene c-Myc. a) Tumor tissues obtained from mice injected with shControl or shSTBD1 HCT116 cells were used to assess the expression of c-Myc by IHC. Images were captured using an Olympus microscope, and integrated density was determined using Image J. Scale bars, 100 µm. **b**) Tumor tissues obtained from mice injected with shControl or shSTBD1 HCT116 cells were extracted by RIPA buffer, and the expression of STBD1, c-Myc and tubulin was determined by immunoblotting. **c**) Tumor tissues obtained from mice injected with shSTBD1/plvx neo, shSTBD1/WT, or shSTBD1/W203C HCT116 cells were used to assess the expression of c-Myc by IHC. Images were captured using an Olympus microscope, and integrated density was determined using Image J. Scale bars, 100 µm. **d**) The same tumor tissues in (c) were extracted by RIPA buffer, and the expression of STBD1, c-Myc and GAPDH was determined by immunoblotting. Statistical data are presented as mean ± SD. Statistical comparisons were

performed using unpaired t-test. *** $p < 0.001$, ** $p < 0.01$, * $p < 0.05$.

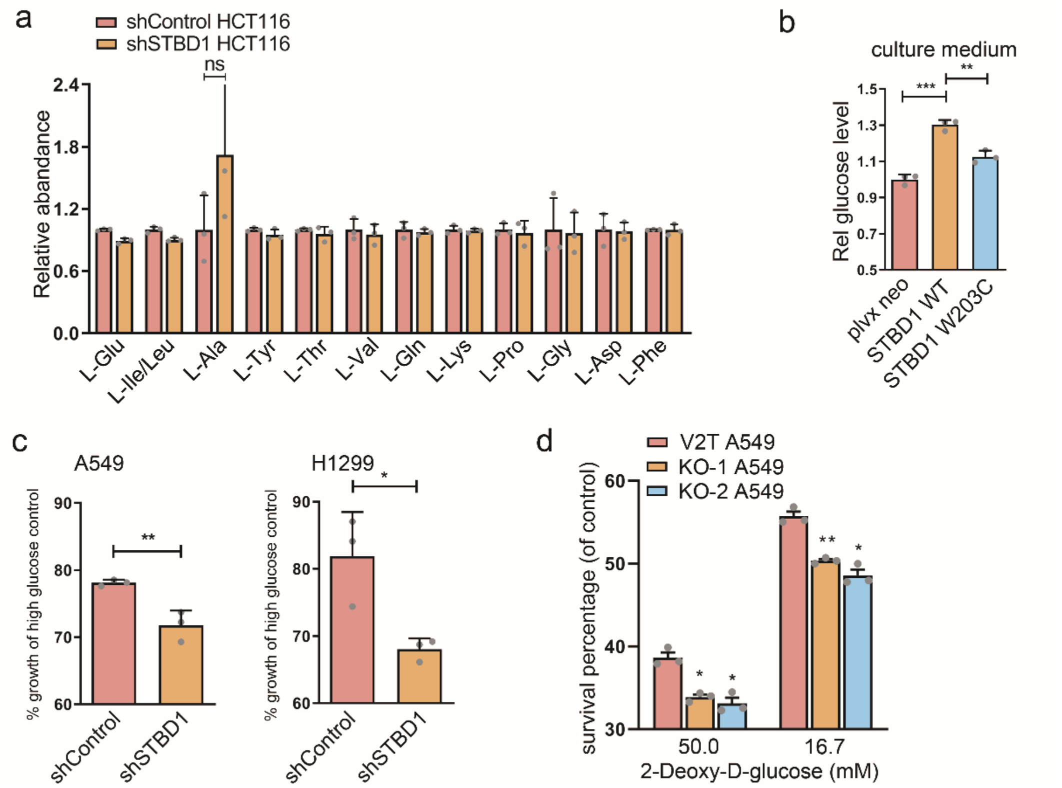

Supplementary Figure 10 | Mutation or deletion of STBD1 promotes the dependence of cancer cells on glycolysis. a) Amino acid accumulation in shControl and shSTBD1 HCT116 cells was measured using LC-MS/MS. Graphical representations of concentrations of amino acid in shControl and shSTBD1 cells. **b**) Control A549 cells and A549 cells stably overexpressing STBD1 WT or STBD1 W203C were cultured for 48 h, then the medium was collected to determine relative glucose concentrations. **c**) shControl and shSTBD1 A549 cells or shControl and shSTBD1 H1299 cells were grown in low glucose medium for 72 h. Cell viability was then assessed using the MTT assay, and were normalized to that of cells grown in high glucose medium. **d**) Control A549 cells and two STBD1-knockout cell lines were treated with different concentrations of 2-DG for 48 h. The cell survival rate in each group was evaluated by the MTT assay, and normalized to that of control group (0 mM). Experiments (b-d) were performed in triplicate. Statistical data were presented as mean ± SD. Statistical comparisons were performed using unpaired t-test. *** p <0.001, ** p <0.01, * p <0.05, ns (not significant) p >0.05.

Supplementary Note 1. Additional comparisons of pLIRm and other existing methods

In iLIR [1], there were 27 known LC3-interacting region (LIR) motifs in 26 LIR-containing proteins (LIRCPs) taken as the positive data for training. For a justified comparison, 227 LMP(7, 7) items around other putative canonical LIR (cLIR) motifs in the same proteins are taken as negative data. Using the same data set, we re-train a model for pLIRm, and the self-consistency validation is performed, with an area under the curve (AUC) value of 0.9790 (Supplementary Fig. 1a). This performance is slightly better than iLIR (0.9584) as well as other methods. Also, the remaining 79 LIRCPs with 100 positive LIR motifs and 704 negative LMP(7, 7) items are not used for training and taken as an independent data set. On this data set, pLIRm is still slightly better than iLIR (0.7610 *vs*. 0.7372) and other methods (Supplementary Fig. 1b). The results demonstrate that our algorithm is not much superior against other existing methods on the small training data set. Also, the large difference between the AUC values of the self-consistency validation and validation on the independent data set indicates that all computational models of pLIRm and other existing methods are highly over-fitted due to the data limitation for training. Undoubtedly, a much larger training data set can both improve the prediction accuracy and robustness.

Using iLIR[1], two databases including iLIR database[2] and iLIR@viral database[3] were constructed to maintain predicted LIRCPs in several model eukaryotes and viruses, respectively. Because no threshold was set in iLIR for predicting LIRCPs, here we estimate its performance using our benchmark data set, containing 127 positive motifs and 931 negative motifs from 105 LIRCPs.

Both sensitivity (*Sn*) and specificity (*Sp*) are calculated under different iLIR scores, and *Sp* = 90.0% is adopted as the threshold (iLIR score ≥ 13). Using the same threshold of pLIRm derived from the 10-fold cross-validation (*Sp* = 90.0%, pLIRm score ≥ -5.65), we predict potential LIRs in reserved LIRCPs from iLIR[2] and iLIR@viral databases[3], respectively. It can be found that only a moderate proportion of iLIR-predicted hits can be covered by pLIRm (Supplementary Fig. 1c). We carefully check the results, and find that pLIRm and iLIR exhibit different superiority and weakness to predict LIRs. For example, human ATG4B was experimentally identified with two LIR motifs, $_{8}$YDTL$_{11}$ and $_{388}$FEIL$_{391}$ [1, 4, 5, 6]. pLIRm can only correctly recognize the former, whereas iLIR can only predict the latter as the positive hit (Supplementary Fig. 1d). For human TOLLIP with two known LIR motifs, $_{133}$WTHI$_{136}$ and $_{151}$WYSL$_{154}$ [7], pLIRm can correctly predict both LIR motifs as positive hits, whereas iLIR can only recognize the $_{151}$WYSL$_{154}$ motif (Supplementary Fig. 1d). In addition, human ATG2A has only one experimentally identified iLIR motif $_{1362}$FCIL$_{1365}$ [5]. iLIR cannot correctly recognize this motif (Supplementary Fig. 1d). In conclusion, pLIRm use a much large data set for training, and exhibit a higher accuracy than iLIR.

Supplementary References

1. Kalvari I, *et al.* iLIR: A web resource for prediction of Atg8-family interacting proteins. *Autophagy* **10**, 913-925 (2014).

2. Jacomin AC, Samavedam S, Promponas V, Nezis IP. iLIR database: A web resource for LIR motif-containing proteins in eukaryotes. *Autophagy* **12**, 1945-1953 (2016).

3. Jacomin AC, Samavedam S, Charles H, Nezis IP. iLIR@viral: A web resource for LIR motif-containing proteins in viruses. *Autophagy* **13**, 1782-1789 (2017).

4. Rogov V, Dotsch V, Johansen T, Kirkin V. Interactions between autophagy receptors and ubiquitin-like proteins form the molecular basis for selective autophagy. *Molecular cell* **53**, 167-178 (2014).

5. Johansen T, Lamark T. Selective Autophagy: ATG8 Family Proteins, LIR Motifs and Cargo Receptors. *Journal of molecular biology* **432**, 80-103 (2020).

6. Birgisdottir AB, Lamark T, Johansen T. The LIR motif - crucial for selective autophagy. *Journal of cell science* **126**, 3237-3247 (2013).

7. Kirkin V, Rogov VV. A Diversity of Selective Autophagy Receptors Determines the Specificity of the Autophagy Pathway. *Molecular cell* **76**, 268-285 (2019).
