# Supplementary material for: Model-based analysis uncovers mutations altering autophagy selectivity in human cancer
Source: Nat Commun. 2021 May 31;12:3258. doi: 10.1038/s41467-021-23539-5 (PMC8166871; doi:10.1038/s41467-021-23539-5)
Supplement: Supplementary file 3 — Description of Additional Supplementary Files [file 41467_2021_23539_MOESM3_ESM.pdf]

Description of Additional Supplementary Files

File Name: Supplementary Data 1

Description: From the literature and public data resources, we constructed a high-quality benchmark data set, containing 127 experimentally identified LIR motifs in 105 LIRCPs.

File Name: Supplementary Data 2

Description: The leave-one-out (LOO) validation and 4-, 6-, 8- and 10-fold cross-validations are performed for pLIRm. We calculate 6 measurements, including accuracy (*Ac*), sensitivity (*Sn*), specificity (*Sp*), positive predictive value (*PPV*), negative predictive value (*NPV*) and Mathew correlation coefficient (*MCC*).

File Name: Supplementary Data 3

Description: Using iCAL, we in total identified 222 potential LAMs that significantly change 172 cLIR motifs in 148 LIRCPs, including 60 Type I and 162 Type II LAMs.

File Name: Supplementary Data 4

Description: Survival analyses of the association between the TCGA data and clinical outcomes are performed for each layer of the omics data in both pan-cancer and individual cancer levels, including cancer single nucleotide variants (SNVs), RNA sequencing (RNA-seq) and DNA methylation. The log-rank test, SNV: p-value < 0.05; RNA expression: p-value < 10^{-4}; DNA methylation: p-value < 10^{-4}.

File Name: Supplementary Data 5

Description: Information about mutations of LIR used in this experiment.

File Name: Supplementary Data 6

Description: RNA-seq analysis of shControl HCT116 cells and shSTBD1 HCT116 cells. Three biological replicates were conducted for each type of cells. Differentially expressed genes were

detected based on | TPM fold change | > 1 and *p*-value < 10^{-5} (Marked in grey). Cancer hallmark proteins were detected based *p*-value < 10^{-3}.

File Name: Supplementary Data 7

Description: RNA-seq analysis of shSTBD1 HCT116 cells transfected with vector (plvx neo), shSTBD-resistant STBD1 WT or STBD1 W203C. Three biological replicates were conducted for each type of cells. Differentially expressed genes between shSTBD1 and shControl, or STBD1 W203C and STBD1 WT, or STBD1 WT and vector (plvx neo) were detected based on | TPM fold change | > 1 and *p*-value < 0.05.

File Name: Supplementary Data 8

Description: Overview of metabolites identified in each sample by targeted metabolomic profiling and isotope tracing analysis.

File Name: Supplementary Data 9

Description: Primers used in qRT-PCR.
