# Supplementary material for: Model-based analysis uncovers mutations altering autophagy selectivity in human cancer
Source: Nat Commun. 2021 May 31;12:3258. doi: 10.1038/s41467-021-23539-5 (PMC8166871; doi:10.1038/s41467-021-23539-5)
Supplement: Supplementary file 13 — Reporting summary [file 41467_2021_23539_MOESM13_ESM.pdf]

nature research

Corresponding author(s): Yu Xue and Da Jia

Last updated by author(s): Mar 29, 2021

Reporting Summary

Nature Research wishes to improve the reproducibility of the work that we publish. This form provides structure for consistency and transparency in reporting. For further information on Nature Research policies, see our Editorial Policies and the Editorial Policy Checklist.

Statistics

For all statistical analyses, confirm that the following items are present in the figure legend, table legend, main text, or Methods section.

n/a	Confirmed	
☐	☒	The exact sample size (*n*) for each experimental group/condition, given as a discrete number and unit of measurement
☐	☒	A statement on whether measurements were taken from distinct samples or whether the same sample was measured repeatedly
☐	☒	The statistical test(s) used AND whether they are one- or two-sided<br>*Only common tests should be described solely by name; describe more complex techniques in the Methods section.*
☒	☐	A description of all covariates tested
☐	☒	A description of any assumptions or corrections, such as tests of normality and adjustment for multiple comparisons
☐	☒	A full description of the statistical parameters including central tendency (e.g. means) or other basic estimates (e.g. regression coefficient) AND variation (e.g. standard deviation) or associated estimates of uncertainty (e.g. confidence intervals)
☐	☒	For null hypothesis testing, the test statistic (e.g. *F*, *t*, *r*) with confidence intervals, effect sizes, degrees of freedom and *P* value noted<br>*Give P values as exact values whenever suitable.*
☒	☐	For Bayesian analysis, information on the choice of priors and Markov chain Monte Carlo settings
☒	☐	For hierarchical and complex designs, identification of the appropriate level for tests and full reporting of outcomes
☐	☒	Estimates of effect sizes (e.g. Cohen's *d*, Pearson's *r*), indicating how they were calculated

Our web collection on statistics for biologists contains articles on many of the points above.

Software and code

Policy information about availability of computer code

Data collection	Software for confocal microscopy-Olympus FV10-ASW 2.1 Viewer.<br>Quantification of western analysis-NIH Image J v_1.8.0<br>For RNA-Seq data, sequencing were performed on an Illumina Novaseq 6000<br>ATG proteins and autophagy regulators in Homo sapiens were taken from THANATOS (http://thanatos.biocuckoo.org/).
Data analysis	Statistical analysis of data from glycogen assay and confocal microscopy, MTT, colony formation-Graphpad Prism 7<br>For Pearson's correlation coefficients-NIH Image J v_1.8.0<br>Differential expression analysis of two groups (three biological replicates)-DESeq2 1.22.2 and DESeq2 1.30.0 (https://bioconductor.org/packages/release/bioc/html/DESeq2.html).<br>RNA-Seq reads were aligned and quantified using RSEM v1.3.1 and STAR v2.7.1a<br>KEGG pathways enrichment analysis-Rpackage clusterProfiler v3.10.1 (https://bioconductor.org/packages/release/bioc/html/clusterProfiler.html).<br>The reads counts mapped to each gene were counted using R package GenomicAlignments v1.18.1 (https://bioconductor.org/packages/release/bioc/html/GenomicAlignments.html).<br>Data analysis and quantitation were performed by the software TraceFinder 3.2 (Thermo Fisher).<br>The Kaplan-Meier survival curves were illustrated by the R package survminer v0.4.6 with the function of "ggsurvplot" (http://www.sthda.com/english/rpkgs/survminer/).<br>Network was constructed and visualized with Cytoscape 3.7.2 software package

For manuscripts utilizing custom algorithms or software that are central to the research but not yet described in published literature, software must be made available to editors and reviewers. We strongly encourage code deposition in a community repository (e.g. GitHub). See the Nature Research guidelines for submitting code & software for further information.

Data

Policy information about availability of data

All manuscripts must include a data availability statement. This statement should provide the following information, where applicable:

- Accession codes, unique identifiers, or web links for publicly available datasets
- A list of figures that have associated raw data
- A description of any restrictions on data availability

The RNA-seq data is deposited into GEO with the accession code GSE173273 and GSE173274 (https://www.ncbi.nlm.nih.gov/geo/query/acc.cgi?acc=GSE173273; https://www.ncbi.nlm.nih.gov/geo/query/acc.cgi?acc=GSE173274). The source code and dataset of pLIRm and pLAM has been uploaded to Github(https://github.com/BioCUCKOO/pLIRm-pLAM). Human cancer mutations were obtained from the TCGA data portal (https://portal.gdc.cancer.gov/, level 4 data, in May, 2018). We downloaded all simple somatic mutations of International Cancer Genome Consortium (ICGC) release 28 from ICGC data portal (https://dcc.icgc.org/releases/release_28/Projects, in November 2019). We downloaded census cancer mutations from Catalogue of Somatic Mutations in Cancer (COSMIC) in file 'CosmicMutantExportCensus.tsv.gz' from COSMIC website (https://cancer.sanger.ac.uk/cosmic/download, in Jan 2019). Human cancer mutations (*Mutation_Packager_Calls.Level_3.*), mRNA expression levels (*.mRNAseq_Preprocess.Level_3.*), DNA methylation profiles (*.Merge_methylation_*.Level_3.*), and clinical outcomes (*.Merge_Clinical.Level_1.*) were obtained from BROAD Institute (http://gdac.broadinstitute.org/runs/stddata__latest/). 127 experimentally-identified LIR motifs in 105 LIRCPs in Homo sapiens and Saccharomyces cerevisiae collected From the literature are provided in Supplementary Data 1. 222 potential LAMs that significantly change 172 cLIR motifs in 148 LIRCPs identified using pLIRm are provided in Supplementary Data 3. Survival analyses of the association between the TCGA data and clinical outcomes are performed for each layer of the omics data in both pan-cancer and individual cancer levels, including cancer single nucleotide variants (SNVs), RNA sequencing (RNA-seq) and DNA methylation are provided in Supplementary Data 4. RNA-seq data are provided in Supplementary Data 6 and 7. Raw metabolomics data sets are included in Supplementary Data 8. All other data supporting the findings of this study are available from the corresponding authors on reasonable request.

Field-specific reporting

Please select the one below that is the best fit for your research. If you are not sure, read the appropriate sections before making your selection.

[x] Life sciences [ ] Behavioural & social sciences [ ] Ecological, evolutionary & environmental sciences

For a reference copy of the document with all sections, see nature.com/documents/nr-reporting-summary-flat.pdf

Life sciences study design

All studies must disclose on these points even when the disclosure is negative.

Sample size	Sample size calculations were not preformed. All cell biology experiments were performed using at least 3 independent biological repeats. Analysis of cell-based phenotypes by confocal microscope was performed on in excess of 30 cells per experimental conditions. Tumor Xenograft were established on 9 BALB/c nude mice.
Data exclusions	No data was excluded from any of the analyses reported in this study.
Replication	All experiments were performed using at least 3 independent biological repeats. All experiments shown were reproducible.
Randomization	Experimental groups were assembled such that controls and positive/negative experimental conditions were generated, processed and analysed in parallel.
Blinding	Blinding was not used in this study.

Reporting for specific materials, systems and methods

We require information from authors about some types of materials, experimental systems and methods used in many studies. Here, indicate whether each material, system or method listed is relevant to your study. If you are not sure if a list item applies to your research, read the appropriate section before selecting a response.

Materials & experimental systems

n/a	Involved in the study	
[ ]	[x]	Antibodies
[ ]	[x]	Eukaryotic cell lines
[x]	[ ]	Palaeontology and archaeology
[ ]	[x]	Animals and other organisms
[x]	[ ]	Human research participants
[x]	[ ]	Clinical data
[x]	[ ]	Dual use research of concern

Methods

n/a	Involved in the study	
[x]	[ ]	ChIP-seq
[x]	[ ]	Flow cytometry
[x]	[ ]	MRI-based neuroimaging

Antibodies

Antibodies used	All antibodies used in this study were obtained from commercial resources: STBD1 (proteintech, 11842-1-AP), GFP (proteintech, 50430-2-AP), Tubulin (proteintech, 11224-1-AP), mCherry (proteintech, 26765-1-AP), GAPDH (proteintech, 10494-1-AP), LC3B (Novus, NB100-2220), c-Myc (proteintech, 10828-1-AP), Flag (SAB, T519), β-actin (proteintech, 60008-1-Ig), AKT1 (CST, 2938), Ki67 (Servicebio, GB13030-2), NFKB1 (CST, 3035)Glycogen (IV58B6) (Baba, 1993), Goat anti-Rabbit IgG Secondary Antibody HRP conjugated (SAB, L3012-2), Goat anti-Mouse IgG Secondary Antibody HRP conjugated (SAB, L3032-2)., and Alexa Fluor 647 goat anti-mouse IgG(Jackson ImmunoResearch).
Validation	Antibodies validation was either through the manufacturers validation sheet (see detailed information above for the precise manufacturer and individual antibody ID) or published validation by other research groups (see reference list associated with the manuscript).

Eukaryotic cell lines

Policy information about cell lines

Cell line source(s)	HEK293T, H1299, A549, HCT116, HeLa, A2780, MDA-MB-468, MGC803, HGC27, HT29, LO2, HepG2, MDA-MB-221 and H460 were obtained from the American Type Culture Collection (ATCC) and Cell Bank of Chinese Academic of Sciences (Shanghai, China), respectively.
Authentication	Authentication was from the ATCC and Cell Bank of Chinese Academic of Sciences. We have authenticated the cell lines HCT116, H1299, A549 and HGC27.
Mycoplasma contamination	The HEK293T and parental cells, including HCT116, H1299, A549 and HGC27 cell lines, were mycoplasma tested, which revealed no contamination.
Commonly misidentified lines (See ICLAC register)	No commonly misidentified cell lines were used.

Animals and other organisms

Policy information about studies involving animals; ARRIVE guidelines recommended for reporting animal research

Laboratory animals	5-week-old BALB/c nude mice (Charles River)
Wild animals	The study did not involve wild animals.
Field-collected samples	This study did not use field-collected samples.
Ethics oversight	The animal welfare and experiments conformed to the guidelines for care and use of laboratory animals and were performed according to the guidelines and approval of the Animal Investigation Committee of the West China Second University Hospital, Sichuan University.

Note that full information on the approval of the study protocol must also be provided in the manuscript.
